# Supplementary material for: MYCN-driven fatty acid uptake is a metabolic vulnerability in neuroblastoma
Source: Nat Commun. 2022 Jun 28;13:3728. doi: 10.1038/s41467-022-31331-2 (PMC9240069; doi:10.1038/s41467-022-31331-2)
Supplement: Supplementary file 1 — Supplementary Information [file 41467_2022_31331_MOESM1_ESM.pdf]

Supplementary Information for MYCN-driven Fatty Acid Uptake is a Metabolic Vulnerability in Neuroblastoma

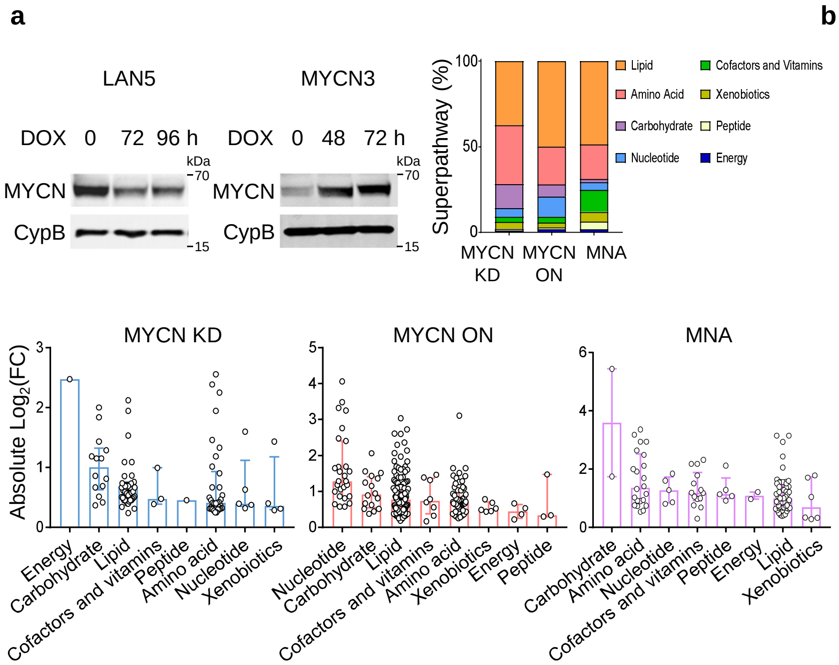

b

Superpathway	Subpathway	Metabolite	KD	ON	MNA
Lipid	Diacylglycerol	diacylglycerol (14:0/18:1, 16:0/16:1) [2]*	0.77	2.31	2.96
		diacylglycerol (16:1/18:2 [2], 16:0/18:3 [1])*	0.76	1.8	7.67
		palmitoyl-palmitoyl-glycerol (16:0/16:0) [2]*	0.73	1.24	1.16
		palmitoyl-oleoyl-glycerol (16:0/18:1) [1]*	0.76	2.64	5.41
		palmitoyl-oleoyl-glycerol (16:0/18:1) [2]*	0.94	1.72	1.64
		palmitoyl-linoleoyl-glycerol (16:0/18:2) [2]*	1.02	1.58	2.43
		palmitoyl-arachidonoyl-glycerol (16:0/20:4) [2]*	0.96	1.16	1.03
		oleoyl-linoleoyl-glycerol (18:1/18:2) [2]	0.89	1.71	2.47
		stearoyl-arachidonoyl-glycerol (18:0/20:4) [1]*	0.85	1.11	0.91
		stearoyl-arachidonoyl-glycerol (18:0/20:4) [2]*	1.11	0.8	0.74
Lipid	Phospholipid Metabolism	choline	1.32	1.03	0.93
		choline phosphate	1.01	0.58	0.93
		cytidine 5'-diphosphocholine	1.48	0.56	0.7
		glycerophosphorylcholine (GPC)	2.38	0.64	0.63
		phosphoethanolamine	1.48	1.3	0.68
		cytidine-5'-diphosphoethanolamine	1.15	0.87	0.61
		glycerophosphoethanolamine	1.35	0.82	0.62
		glycerophosphoserine*	1.39	1.28	0.87
		glycerophosphoinositol*	1.02	1.51	0.88
		trimethylamine N-oxide	1.82	1.2	1.5
Amino Acid	Glutamate Metabolism	glutamate	1.23	0.74	0.67
		glutamine	1.37	0.32	0.81
		N-acetylglutamate	0.97	0.89	0.59
		N-acetylglutamine	1.17	0.74	0.69
		4-hydroxyglutamate	1.16	1.31	0.29
		glutamate, gamma-methyl ester	1	0.46	0.34
		pyroglutamine*	0.99	1.63	0.93
		N-acetyl-aspartyl-glutamate (NAAG)	0.95	1.77	0.72
		beta-citrylglutamate	1.41	0.64	0.61
		S-1-pyrroline-5-carboxylate	0.82	0.89	1.12
Lipid	Endocannabi-noid	oleoyl ethanolamide	0.74	1.11	1.04
		palmitoyl ethanolamide	0.98	1.25	0.95
		stearoyl ethanolamide	1.1	0.95	1.47
		N-oleoyltaurine	0.71	0.77	1.63
		N-stearoyltaurine	0.82	1.17	1.26
		N-palmitoyltaurine	0.66	0.62	0.79
		palmitoleoyl ethanolamide*	0.83	1.9	N/A
Amino Acid	Methionine, Cysteine, SAM and Taurine Metabolism	methionine	1.1	0.82	0.97
		N-acetylmethionine	0.91	0.63	0.52
		N-formylmethionine	0.95	1.05	0.69
		S-methylmethionine	1.16	0.91	0.71
		methionine sulfone	1.2	1.03	0.92
		methionine sulfoxide	1.25	1.1	0.53
		N-acetylmethionine sulfoxide	1	0.54	1.61
		S-adenosylmethionine (SAM)	1.06	0.83	0.66
		S-adenosylhomocysteine (SAH)	1.04	0.93	1.71
		homocysteine	1.21	2.4	1.96
		cystathionine	1.33	0.64	0.4
		cysteine	1.33	0.86	0.54
		N-acetylcysteine	1	0.41	0.68
		cystine	1	0.88	0.76
		cysteine sulfinic acid	1	0.77	0.72
		hypotaurine	1.28	1.13	0.56
		taurine	1.24	1.17	1.34
		N-acetyltaurine	1.24	1.91	1.11
		3-sulfo-L-alanine	1	1.04	2.01
Lipid	Phosphatidyl-choline (PC)	1,2-dipalmitoyl-GPC (16:0/16:0)	1.36	0.85	0.79
		1-palmitoyl-2-stearoyl-GPC (16:0/18:0)	1.43	0.62	0.63
		1-palmitoyl-2-oleoyl-GPC (16:0/18:1)	1.14	0.95	0.92
		1-palmitoyl-2-arachidonoyl-GPC (16:0/20:4n6)	1.19	0.71	0.94
		1-stearoyl-2-oleoyl-GPC (18:0/18:1)	1.16	0.97	0.91
		1,2-dioleoyl-GPC (18:1/18:1)	1.16	1.14	1.11
		1,2-dilinoleoyl-GPC (18:2/18:2)	1	1.11	0.68
		1-stearoyl-2-arachidonoyl-GPC (18:0/20:4)	1.36	0.49	0.93
		1-linoleoyl-2-arachidonoyl-GPC (18:2/20:4n6)*	1.16	0.75	0.64

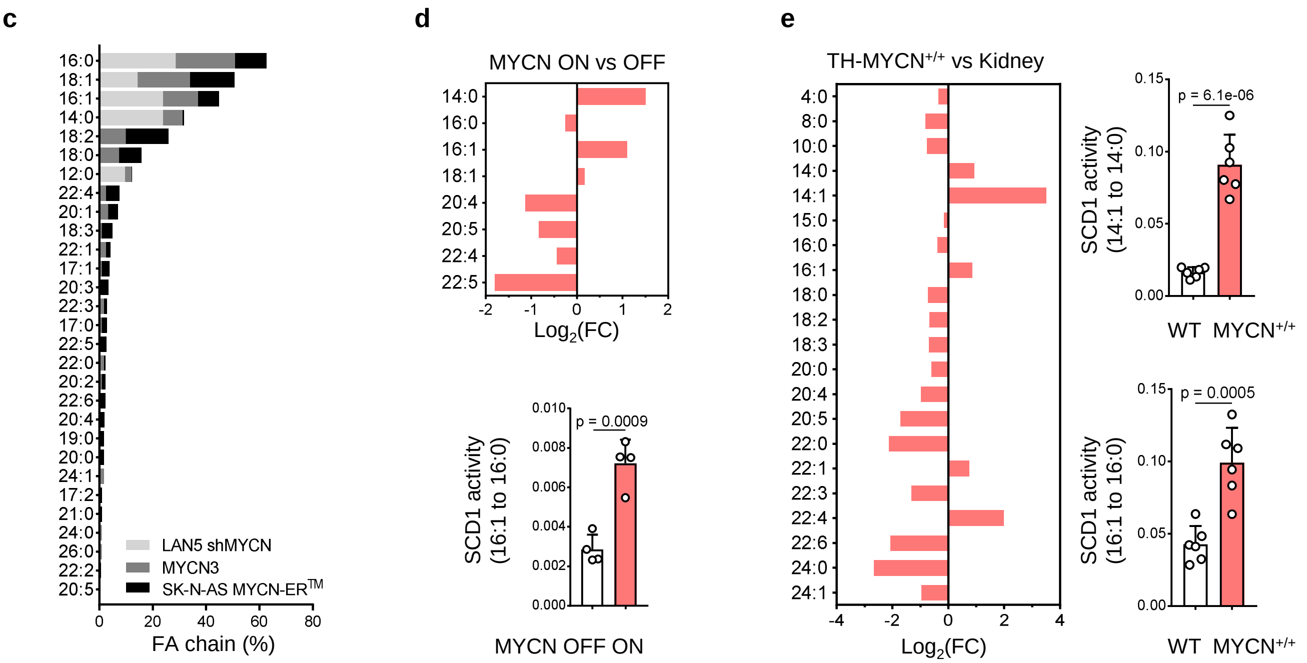

Supplementary Figure 1

Supplementary Fig. 1. MYCN deregulates lipid metabolism. a. Top left, MYCN protein expression in LAN5 shMYCN (0–96 h) and MYCN3 (0–72 h) cells (1 μg/mL DOX). CypB served as a protein loading control. Representative blots from three independent experiments are shown. Top right, percentage of differential metabolites (p≤0.05) in superpathways for the three comparison groups. Data are presented as a stacked bar graph. Bottom, absolute log_2(FC) of differential metabolites (p≤0.05) in superpathways for the three comparison groups. Data are presented as the median with interquartile range. Each group contains 4 biological replicates. One-way ANOVA or Welch's two-sample t-test was used to identify differential metabolites between groups. **b.** Changes in representative metabolites in NB cells and primary tumors (raw data in Supplementary Data 2). Comparison groups: KD=MYCN KD 72 h vs. CTRL; ON=MYCN-ON 72 h vs. MYCN-OFF; MNA=MNA vs. non-MNA. Red/blue: upregulated/downregulated (p≤0.05); Light red/light blue: upregulated/downregulated (0.05<p<0.1). One-way ANOVA or Welch's two-sample t-test was used to compare metabolite levels between groups. **c.** FA chain percentage in MYCN-upregulated glycerolipids (FDR<0.25) across LAN5 shMYCN, MYCN3, and SK-N-AS MYCN-ER™ systems. **d.** FA profiling in MYCN3 cells (MYCN-ON 72 h vs. MYCN-OFF). Differential FAs (FDR<0.25) are represented as log_2(FC). SCD1 activity is estimated by the ratio of total 16:1 to 16:0. Mean±SD (n=4); two-sided unpaired t-test. **e.** FA profiling in TH-MYCN$^{+/+}$ tumors and wild-type kidneys. Differential FAs (FDR<0.25) are represented as log_2(FC). SCD1 activity is estimated by the ratios of total 14:1 to 14:0 and 16:1 to 16:0. Mean±SD (n=6); two-sided unpaired t-test. FC=fold change; KD=knockdown; MNA=*MYCN*-amplified; non-MNA=non *MYCN*-amplified. Source data are provided in the Source Data file.

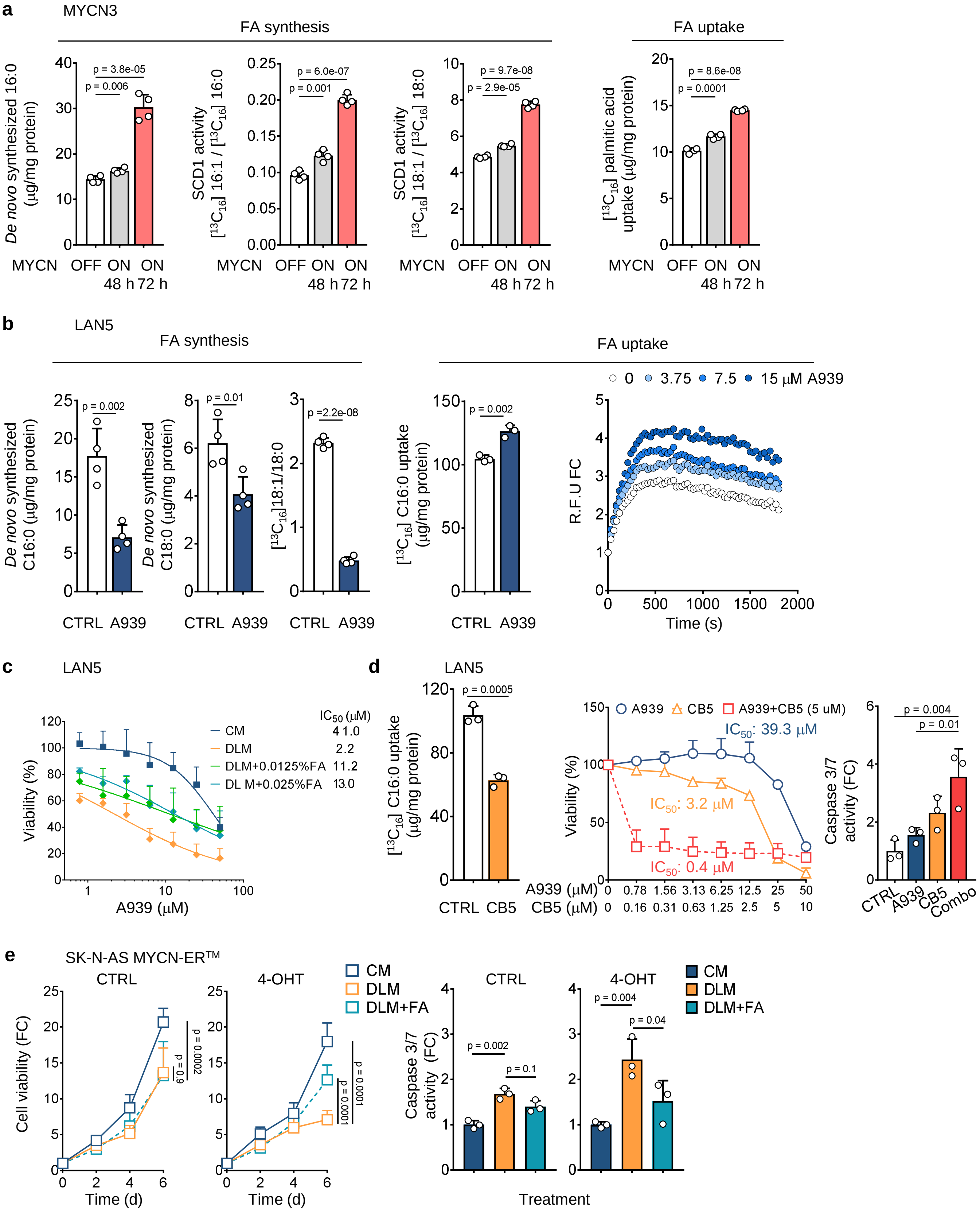

Supplementary Figure 2

Supplementary Fig. 2. MNA NB survival relies highly on FA uptake. a. Stable-isotope tracing of FA synthesis and uptake in MYCN3 cells (0–72 h, 1 µg/mL DOX). Mean±SD (n=4); two-sided unpaired t-test. **b.** Effect of A939572 on FA synthesis and uptake in LAN5 cells. Cells were treated with or without A939572 (25 µM) for 48 h before stable-isotope tracing analysis. Mean±SD (FA synthesis experiments n=3; FA uptake experiments n=3); two-sided unpaired t-test. FA uptake was also evaluated by real-time FA uptake assay (0–15 µM A939572, 15 min). Each dot is the mean of three independent experiments. **c–d.** Effect of blocking FA uptake on cell sensitivity to FA synthesis inhibition. (c) LAN5 cell viability in complete and delipidized media (−/+FAs and A939572) for 72 h. Mean±SD (n=3). IC_{50} values computed by GraphPad Prism (7.01). (d) Left, FA uptake stable-isotope tracing in LAN5 cells with or without CB5 (5 µM for 72 h). Mean±SD (n=3); two-sided unpaired t-test. Middle, LAN5 cell viability after treatment with A939572 (0–50 µM), CB5 (0–10 µM), or A939572 (0–50 µM) + 5 µM CB5 for 72 h. IC_{50} values for A939572 (blue), CB5 (orange), and their combination (red) computed by GraphPad Prism (7.01). Mean±SD (n=3). Right, Caspase 3/7 activity in LAN5 cells treated with CTRL, A939572 (25 µM), CB5 (5 µM), or their combination for 72 h. Mean±SD (n=3); one-way ANOVA with Tukey's multiple comparisons test. **e.** Left, SK-N-AS MYCN-ER™ cell viability in complete media, delipidized media, and delipidized media supplemented with 0.025% FA (−/+ 5 nM 4-OHT, 0–6 days). Mean±SD (n=3); two-way ANOVA with Dunnett's multiple comparisons test. Right, SK-N-AS MYCN-ER™ Caspase 3/7 activity in complete media, delipidized media, and delipidized media supplemented with 0.025% FA (−/+ 5 nM 4-OHT at day 6). Mean±SD (n=3); one-way ANOVA with Dunnett's multiple comparisons test. FC=fold change; A939=A939572; R.F.U.=relative fluorescence unit; CM=complete media; DLM=delipidized media. Source data are provided in the Source Data file.

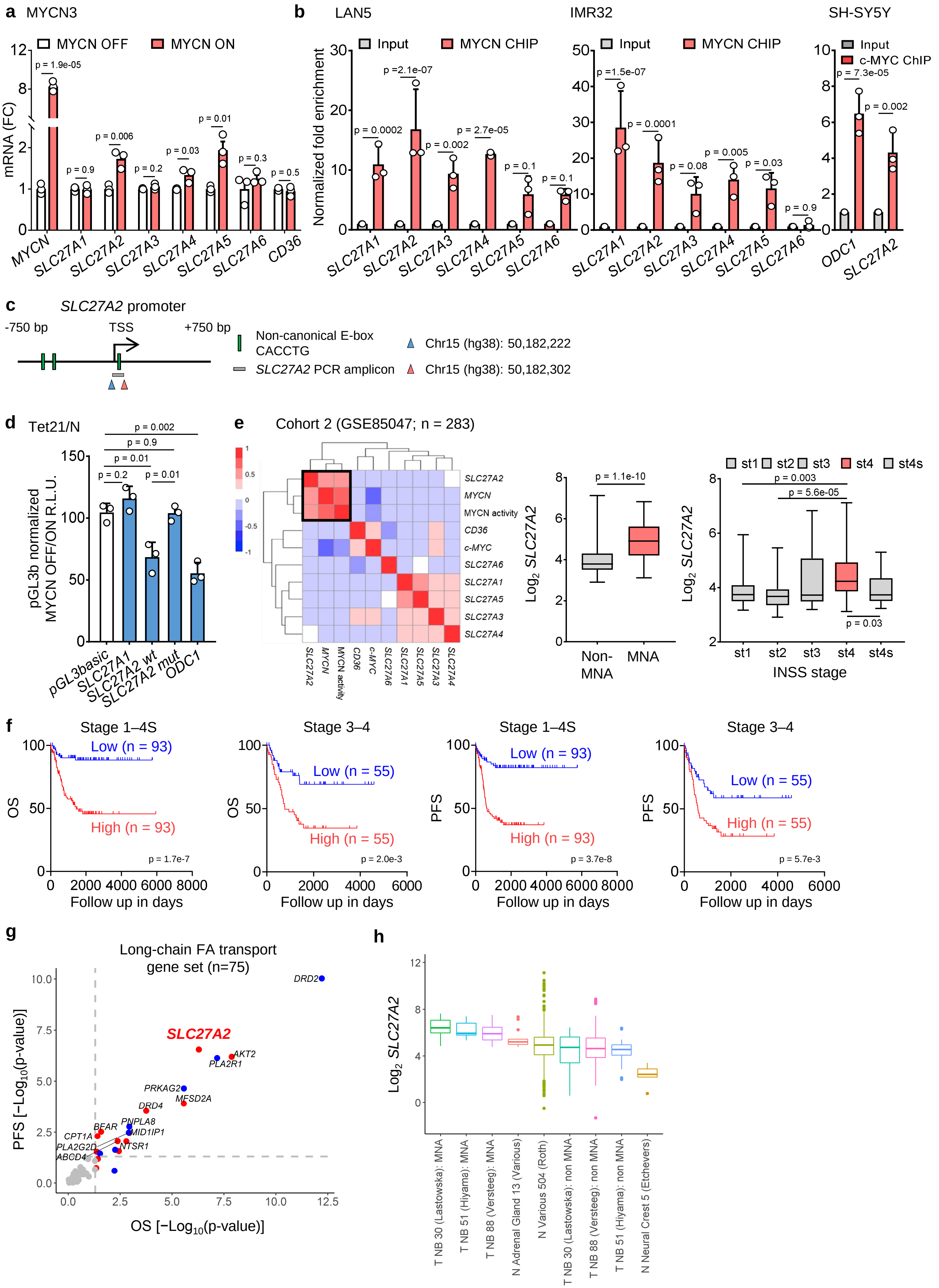

Supplementary Figure 3

Supplementary Fig. 3. MYCN directly upregulates *SLC27A2,* which predicts poor NB survival. a. mRNA expression of *MYCN* and FA transporters (*SLC27A1–6*, *CD36)* in MYCN3 cells (−/+1 µg/mL DOX for 48 h). Mean±SEM (n=3). Two-sided unpaired t-test. **b.** MYCN and c-MYC ChIP-qPCR analyses in NB cells. Mean±SD (n=3). Two-way ANOVA with Sidak's multiple comparisons test. **c.** Diagram of PCR amplification site within the promoter region of *SLC27A2*. TSS=transcription start site; green bar=non-canonical E-box; gray bar=PCR amplicon; blue triangle=chromosome region chr15 (hg38): 50,182,222; red triangle=chromosome region chr15 (hg38): 50,182,302. **d.** *SLC27A1*, *SLC27A2* (wild-type and mutant), and *ODC1* promoter luciferase activities. Results are reported as percentages of MYCN-OFF/ON RLU ratio. pGL3b empty vector and pGL3-ODC1 promoter were used as negative and positive controls, respectively. Mean±SD (n=3); two-sided unpaired t-test. **e.** Gene expression analysis in Cohort 2 (GSE85047). Left, correlation matrix of transporter gene expression, *MYCN* expression/activity, and *c-MYC* expression. Correlations with p-values<0.05 are represented in the heatmap. Red=positive correlation; blue=negative correlation. Middle, *SLC27A2* expression in MNA (n=55) and non-MNA patients (n=222). Two-sided unpaired Welch's t-test. Right, *SLC27A2* expression in stage 1–4S patients (stage 1: n=50; stage 2: n=36; stage 3: n=43; stage 4: n=124; stage 4s: n=27). One-way ANOVA with Tukey's multiple comparisons test. **f–g.** Survival analysis in Cohort 2 (GSE85047). (f) OS and PFS rate for stage 1–4S and stage 3–4 patients with high (top third) or low (bottom third) *SLC27A2* expression. (g) OS and PFS prediction of genes in the long-chain FA transport gene set (GO: 0015909). Log-rank test was used for statistical analysis. Red=high expression associated with poor prognosis (p<0.05); blue=low expression associated with poor prognosis (p<0.05); gray=no significance. **h.** *SLC27A2* expression in NB tumors and normal tissues. Tumor samples were stratified into MNA and non-MNA tumors. Lastowska, GSE13136 (MNA n=10, non-MNA n=20); Hiyama, GSE16237 (MNA n=7, non-MNA n=44); Versteeg, GSE16476 (MNA n=16, non-MNA n=72); Adrenal Gland, SN_ADGL (n=13); Neural Crest, GSE14340 (n=5); Normal Various, GSE7307 (n=504). Box plots indicate median (middle line), 25th and 75th percentiles (box), min and max or 1.5× interquartile range (whisker), as well as outliers (single point). FC=fold change; R.L.U.=relative luminescence unit; OS=overall survival; PFS=progression-free survival. Source data are provided in the Source Data file.

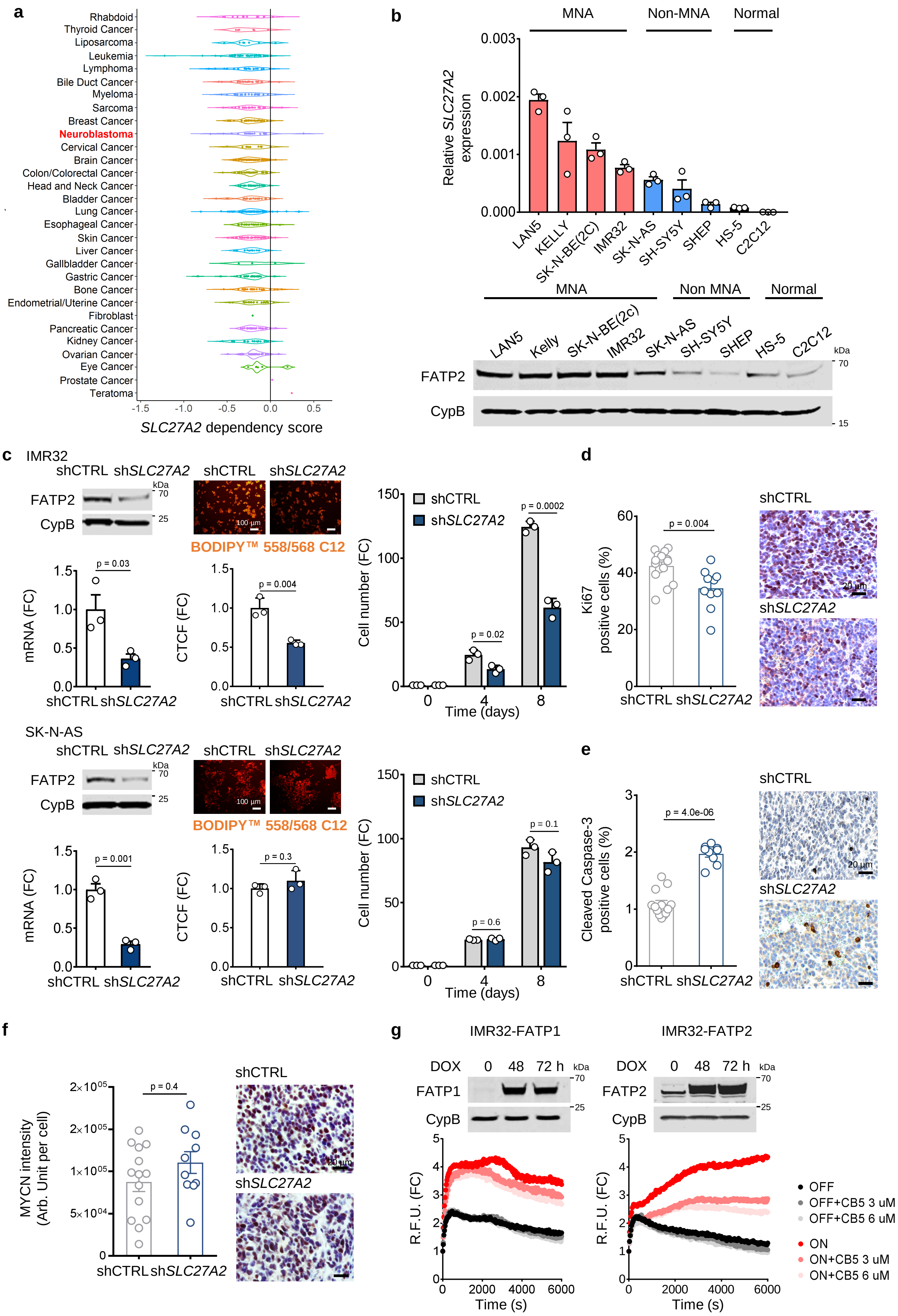

Supplementary Figure 4

Supplementary Fig. 4. *SLC27A2* dependency and expression across multiple NB and normal cell lines. a. *SLC27A2* dependency in multiple cancer types and normal cells. Data were retrieved from the CRISPR (Avana) Public 20Q4V2 dataset and ranked according to the average *SLC27A2* dependency score (low to high). A low score indicates a high dependency on *SLC27A2* for cell survival. **b.** *SLC27A2* mRNA and protein expression in MNA, non-MNA, and normal cells. *GAPDH* and CypB were used as mRNA and protein loading controls, respectively. Mean±SEM (n=3). **c.** Effects of silencing *SLC27A2* on FA uptake and cell growth in MNA IMR32 and non-MNA SK-N-AS cells. Left, *SLC27A2* mRNA and FATP2 protein expression in shCTRL and sh*SLC27A2* cells. Mean±SEM (n=3); two-sided unpaired t-test. Middle, FA uptake in shCTRL and sh*SLC27A2* cells. Cells stained with FA analog BODIPY™ 558/568 C12 and quantified as CTCF by ImageJ2. Mean±SD (n=3); two-sided unpaired t-test. Right, cell growth of shCTRL and sh*SLC27A2* cells at days 4 and 8. Fold change in cell number compared to day 0 is shown. Mean±SD (n=3); two-sided unpaired t-test. **d–f.** Immunohistochemical staining of Ki67 (d), cleaved Caspase-3 (e), and MYCN (f) in shCTRL and sh*SLC27A2* LAN5 orthotopic xenograft tumors. Mean±SEM (shCTRL=15 in [d], 13 in [e], 14 in [f] and sh*SLC27A2*=10 in [d], 9 in [e], 10 in [f]); two-sided unpaired Mann–Whitney test. **g.** FATP protein expression and real-time FA uptake in IMR32 cells with conditional FATP1 or FATP2 overexpression. CypB was used as the protein loading control. Cells were induced by 1 µg/mL DOX for 72 h (FATP1 overexpression) and 48 h (FATP2 overexpression) to reach optimal FA uptake potential. Representative blots from three independent experiments are shown. FA uptake was monitored in the presence of 0, 3, or 6 µM CB5 for 6000 s. Each dot presents the mean value of three independent experiments. R.F.U.=relative fluorescence unit; Arb. Unit=arbitrary unit. Source data are provided in the Source Data file.

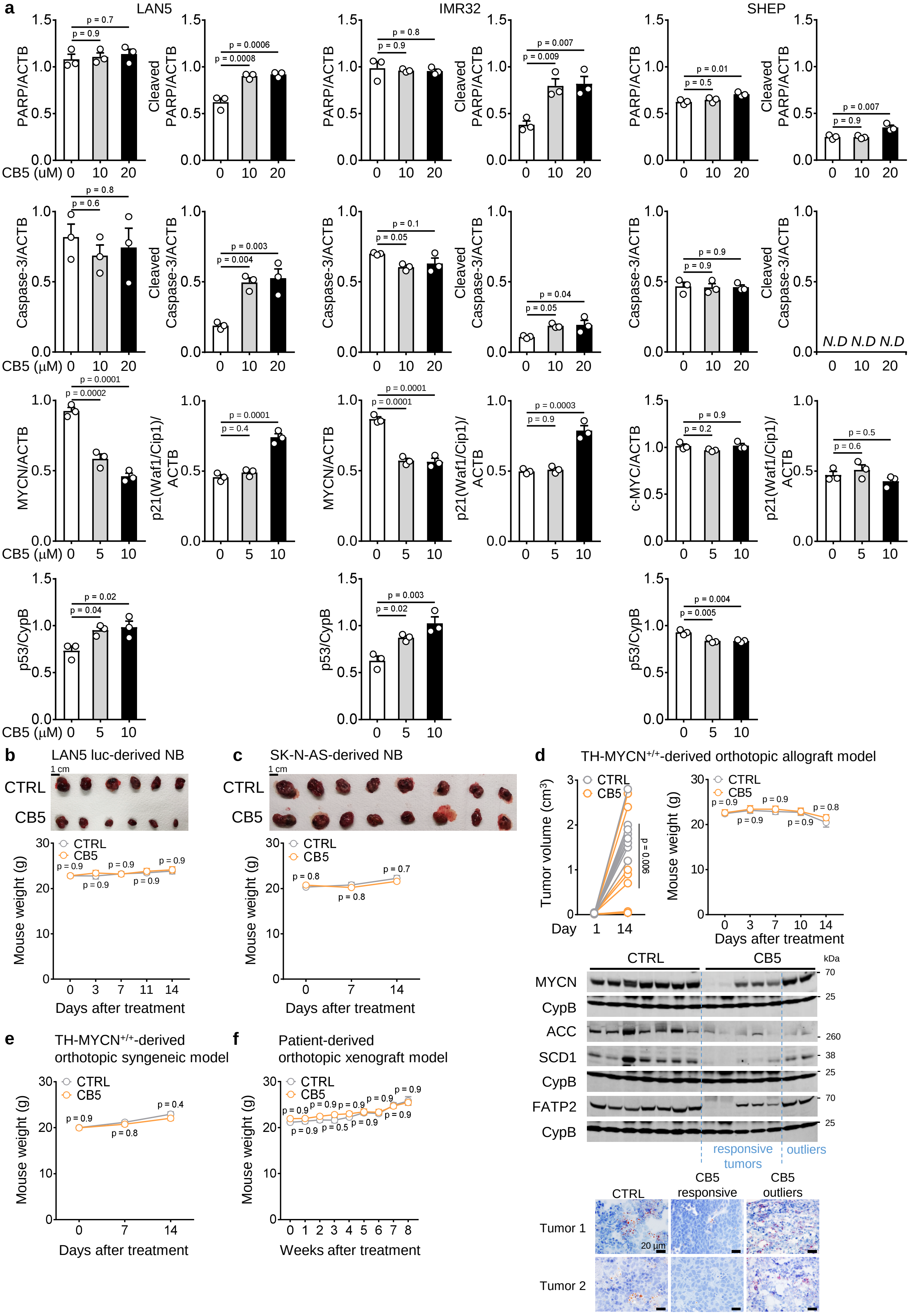

Supplementary Figure 5

Supplementary Fig. 5. Effect of CB5 treatment in NB cells and tumors. a. Quantification of apoptosis and p53, p21, and MYCN (or c-MYC) protein levels in MNA (LAN5 and IMR32) and non-MNA (SHEP) cells upon CB5 treatment (0–20 μM, 16–24 h). Mean±SEM (n=3); one-way ANOVA with Dunnett's multiple comparisons test. *N.D.*=not detected. **b–c.** Tumor images and mouse weight changes during CTRL and CB5 treatment in MNA LAN5-derived orthotopic xenograft model. Mean±SEM (CTRL=5, CB5=6) (b), and non-MNA SK-N-AS-derived orthotopic xenograft model. Mean±SEM (CTRL=8, CB5=8) (c). Two-way ANOVA with Sidak's multiple comparisons test. **d.** Top, tumor volume (CTRL=10, CB5=9) and mouse weight (CTRL=8, CB5=9) changes during CTRL and CB5 treatment in TH-MYCN$^{+/+}$-derived orthotopic allograft model. Mean±SEM . Two-way ANOVA with Sidak's multiple comparisons test. Middle, protein expression of MYCN, SCD1, ACC, and FATP2 in CTRL (n=7) and CB5-treated tumors (n=7). The same samples were run on multiple blots with CypB as loading control on each blot (see uncropped blots in source data). Bottom, Oil Red O staining of intratumoral lipids in CTRL, CB5-responsive tumors, and CB5 non-responsive tumors. **e–f.** Mouse weight changes in (e) TH-MYCN$^{+/+}$-derived orthotopic syngeneic model; mean ± SEM (CTRL=14, CB5=13), and (f) patient-derived orthotopic xenograft model; mean ± SEM (CTRL=8, CB5=9). Two-way ANOVA with Sidak's multiple comparisons test. Source data are provided in the Source Data file.

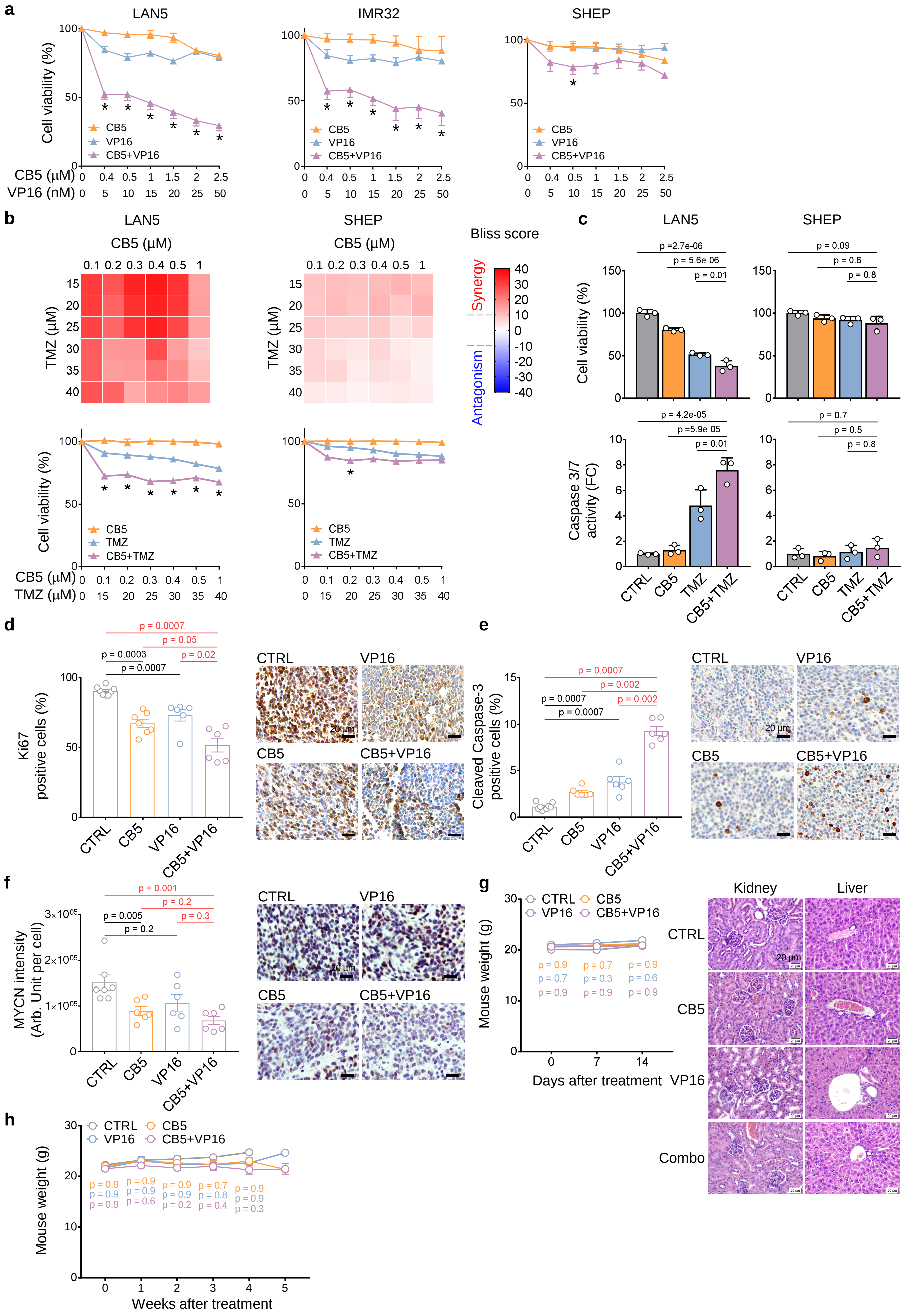

Supplementary Figure 6

Supplementary Fig. 6. Combination therapy in NB cells and tumors. a. MNA (LAN5 and IMR32) and non-MNA (SHEP) cell viability after CB5 (0–2.5 μM), VP16 (0–50 nM), and CB5+VP16 treatment for 72 h (corresponding to Fig. 6a). Mean±SD (n=3); * indicates synergistic effect. **b.** Synergy and cell viability analyses in NB cells treated with CB5 (0–1 μM), TMZ (0–40 μM), and their combination for 72 h. Heatmaps present the mean Bliss score from three independent experiments. Bliss score>10 indicates synergy. Line graph shows cell viability. Mean±SD (n=3); * indicates synergistic effect. **c**. Cell viability and Caspase 3/7 activity of single-drug and combination treatment. LAN5 and SHEP cells were treated with CTRL, CB5 (3 μM), TMZ (60 μM), and their combination for 72 h. Mean±SD (n=3); one-way ANOVA with Tukey's multiple comparisons test. **d–f.** Immunohistochemical staining of Ki67 (d), cleaved Caspase-3 (e), and MYCN (f) in LAN5-derived orthotopic xenograft tumors. Mean±SEM (CTRL=8 in [d,e] and 7 in [f], CB5=7 in [d] and 6 in [e,f], VP16=6, CB5+VP16=6); two-sided unpaired Mann–Whitney test. **g.** Left, mouse weight changes during single-drug (CB5 or VP16) and combination (CB5+VP16) treatment in LAN5-derived orthotopic xenografts. Mean±SEM (CTRL=11, CB5=11, VP16=10, CB5+VP16=12). Two-way ANOVA with Sidak's multiple comparisons test. Right, H&E of normal organs (kidney and liver) in CTRL and treatment groups. **h.** Mouse weight changes during single-drug (CB5 or VP16) and combination (CB5+VP16) treatment in patient-derived orthotopic xenografts. CTRL data not collected at week 5 due to no surviving mice in CTRL group. Mean±SEM (CTRL=12, CB5=10, VP16=10, CB5+VP16=11); Two-way ANOVA with Sidak's multiple comparisons test. FC=fold change. P-value color: Orange (CB5 vs CTRL), Blue (VP16 vs CTRL), Purple (VP16+CB5 vs CTRL). FC=fold change; Arb. Unit=arbitrary unit. Source data are provided in the Source Data file.

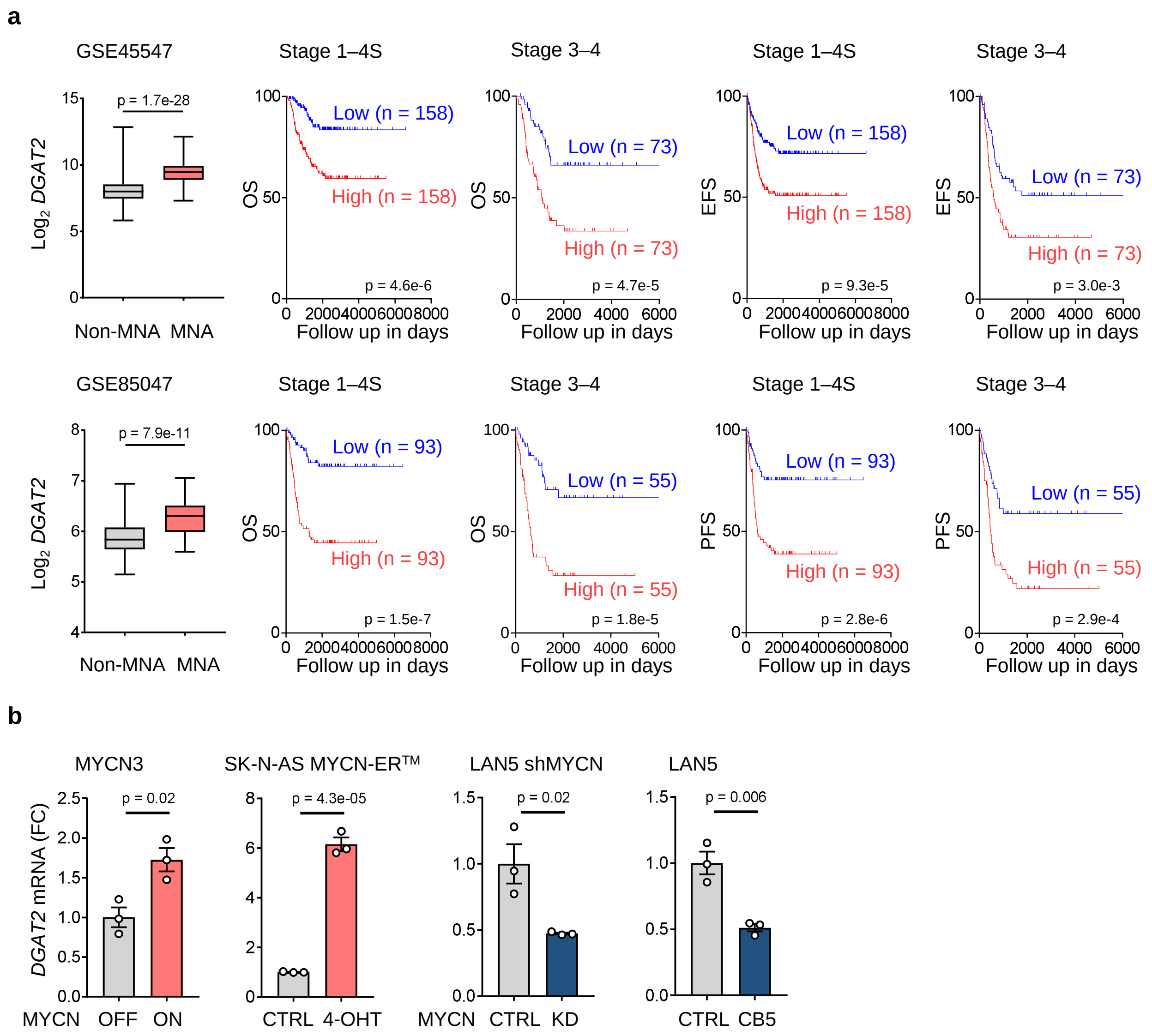

a
GSE45547
p = 1.7e-28
Log2 DGAT2
Non-MNA MNA
Stage 1–4S
Stage 3–4
Low (n = 158)
High (n = 158)
Low (n = 73)
High (n = 73)
OS
EFS
p = 4.6e-6
p = 4.7e-5
p = 9.3e-5
p = 3.0e-3
Follow up in days
GSE85047
p = 7.9e-11
Low (n = 93)
High (n = 93)
Low (n = 55)
High (n = 55)
PFS
p = 1.5e-7
p = 1.8e-5
p = 2.8e-6
p = 2.9e-4
b
MYCN3
SK-N-AS MYCN-ER™
LAN5 shMYCN
LAN5
DGAT2 mRNA (FC)
p = 0.02
p = 4.3e-05
p = 0.02
p = 0.006
MYCN OFF ON
CTRL 4-OHT
MYCN CTRL KD
CTRL CB5

Supplementary Fig. 7. *DGAT2* expression in NB. a. Log_2 *DGAT2* expression in non-MNA and MNA patients (GSE45547, n=649 and GSE85047, n=283). Two-sided unpaired Welch's t-test. Box plots indicate median (middle line), 25th and 75th percentiles (box), as well as min and max (whisker). Survival analysis in GSE45547 and GSE85047. OS and EFS (or PFS) rate of stage 1–4S and stage 3–4 patients with high (top third) or low (bottom third) *DGAT2* expression. Log-rank test was used for statistical analysis. **b.** *DGAT2* mRNA expression in MYCN3 cells (MYCN-ON 72 h vs. MYCN-OFF), SK-N-AS MYCN-ER™ cells (500 nM 4-OHT 48 h vs. CTRL), LAN5 shMYCN cells (MYCN KD 72 h vs. CTRL) and LAN5 cells (20 µM CB5 24 h vs. CTRL). Mean±SEM (n=3); two-sided unpaired t-test. OS=overall survival; EFS=event-free survival; PFS=progression-free survival; FC=fold change. Source data are provided in the Source Data file.
