# Supplementary material for: MYCN-driven fatty acid uptake is a metabolic vulnerability in neuroblastoma
Source: Nat Commun. 2022 Jun 28;13:3728. doi: 10.1038/s41467-022-31331-2 (PMC9240069; doi:10.1038/s41467-022-31331-2)
Supplement: Supplementary file 3 — Description of Additional Supplementary Files [file 41467_2022_31331_MOESM3_ESM.pdf]

Description of Additional Supplementary Files

File Name: Supplementary Data 1
Description: Patient sample information. MNA (n=18) and non-MNA (n=18) NB tumors were provided by the RTSS at TCH.

File Name: Supplementary Data 2
Description: Metabolomics profiling and pathway analysis. Global metabolomics profiling was performed in cell samples (MYCN KD 0, 72, 96 h; MYCN ON 0, 48, 72 h; n=4 per condition) and primary tumor samples (MNA, n=18; non-MNA, n=18). One-way ANOVA was used to compare metabolite levels between groups. Welch's two-sample t-test was used to compare metabolite levels between MNA and non-MNA primary tumors. Red and green shaded cells indicate $p \leq 0.05$; Light red and light green shaded cells indicate $0.05 < p < 0.10$. GSEA-based pathway analysis was shown in the file. FDR<0.25 indicates significantly altered subpathways. One-way ANOVA or Welch's two-sample t-test was used to compare metabolite levels between groups.

File Name: Supplementary Data 3
Description: Lipidomics profiling in NB cells and tumors. Normalized lipid levels in LAN5 shMYCN cells (MYCN KD and CTRL, n=4 each), MYCN3 cells (MYCN OFF and ON, n=4 each), SK-N-AS MYCN-ER™ cells (CTRL and 4-OHT, n=4 each) and NB tumors (shCTRL and sh*SLC27A2*, n=8 each).

File Name: Supplementary Data 4
Description: Real time qPCR, ChIP-qPCR primers and pGL3 promoter cloning oligos. ChIP-qPCR and luciferase reporter activity assay were performed to determine MYCN-mediated transcription of FA transporter genes. The primers and regions of interest of FA transporters (*SLC27A1-6*, *CD36*) and positive control (*ODC1*) were provided in the file.
