# Supplementary material for: MYCN-driven fatty acid uptake is a metabolic vulnerability in neuroblastoma
Source: Nat Commun. 2022 Jun 28;13:3728. doi: 10.1038/s41467-022-31331-2 (PMC9240069; doi:10.1038/s41467-022-31331-2)
Supplement: Supplementary file 8 — Reporting Summary [file 41467_2022_31331_MOESM8_ESM.pdf]

nature portfolio

Corresponding author(s): Eveline Barbieri, MD PhD

Last updated by author(s): 06/06/2022

Reporting Summary

Nature Portfolio wishes to improve the reproducibility of the work that we publish. This form provides structure for consistency and transparency in reporting. For further information on Nature Portfolio policies, see our Editorial Policies and the Editorial Policy Checklist.

Statistics

For all statistical analyses, confirm that the following items are present in the figure legend, table legend, main text, or Methods section.

n/a	Confirmed	
☐	☒	The exact sample size (n) for each experimental group/condition, given as a discrete number and unit of measurement
☐	☒	A statement on whether measurements were taken from distinct samples or whether the same sample was measured repeatedly
☐	☒	The statistical test(s) used AND whether they are one- or two-sided<br>*Only common tests should be described solely by name; describe more complex techniques in the Methods section.*
☒	☐	A description of all covariates tested
☐	☒	A description of any assumptions or corrections, such as tests of normality and adjustment for multiple comparisons
☐	☒	A full description of the statistical parameters including central tendency (e.g. means) or other basic estimates (e.g. regression coefficient) AND variation (e.g. standard deviation) or associated estimates of uncertainty (e.g. confidence intervals)
☐	☒	For null hypothesis testing, the test statistic (e.g. F, t, r) with confidence intervals, effect sizes, degrees of freedom and P value noted<br>*Give P values as exact values whenever suitable.*
☒	☐	For Bayesian analysis, information on the choice of priors and Markov chain Monte Carlo settings
☒	☐	For hierarchical and complex designs, identification of the appropriate level for tests and full reporting of outcomes
☐	☒	Estimates of effect sizes (e.g. Cohen's d, Pearson's r), indicating how they were calculated

Our web collection on statistics for biologists contains articles on many of the points above.

Software and code

Policy information about availability of computer code

Data collection	Microsoft Excel (2013), Odyssey® Application Software 3.0, Analyst TF software 1.8
Data analysis	GSEA (4.0.3), Living Image (4.7.3), Osirix (5.8.5), alpha View-Fluorchem Q software (3.4.0.0), Cytoscape 3.8.2, Agilent mass hunter quantitative software (10.0), GraphPad Prism (7.01), R software (4.0.4), ImageJ2, R2: Genomics Analysis and Visualization Platform (http://r2.amc.nl, no version number)

For manuscripts utilizing custom algorithms or software that are central to the research but not yet described in published literature, software must be made available to editors and reviewers. We strongly encourage code deposition in a community repository (e.g. GitHub). See the Nature Portfolio guidelines for submitting code & software for further information.

Data

Policy information about availability of data

All manuscripts must include a data availability statement. This statement should provide the following information, where applicable:

- Accession codes, unique identifiers, or web links for publicly available datasets
- A description of any restrictions on data availability
- For clinical datasets or third party data, please ensure that the statement adheres to our policy

Five human NB tumor datasets (Kocak, GSE45547[https://www.ncbi.nlm.nih.gov/geo/query/acc.cgi?acc=GSE45547], n=649; NRC, GSE85047[https://www.ncbi.nlm.nih.gov/geo/query/acc.cgi?acc=GSE85047], n=283; Lastowska, GSE13136[https://www.ncbi.nlm.nih.gov/geo/query/acc.cgi?acc=GSE13136], n=30; Hiyama, GSE16237[https://www.ncbi.nlm.nih.gov/geo/query/acc.cgi?acc=GSE16237], n=51; and Versteeg, GSE16476[https://www.ncbi.nlm.nih.gov/geo/query/acc.cgi?acc=GSE16476], n=88) and three normal tissue datasets (Adrenal Gland, SN_ADGL[https://hgserver1.amc.nl/cgi-bin/r2/main.cgi], n=13; Neural Crest, GSE14340[https://www.ncbi.nlm.nih.gov/geo/query/acc.cgi?acc=GSE14340], n=5; and Normal Various, GSE7307[https://www.ncbi.nlm.nih.gov/geo/query/acc.cgi?

March 2021

acc=GSE7307], n=504 including 108 types of normal tissues) were included in this study. They are all publicly available at NCBI Gene Expression Omnibus [https://www.ncbi.nlm.nih.gov/geo] or R2: Genomics Analysis and Visualization Platform [http://r2.amc.nl]. CRISPR (Avana) Public 20Q4V2 [https://depmap.org/portal/download/all/] and the long-chain FA transport geneset (GO: 0015909 [http://amigo.geneontology.org/amigo/term/GO:0015909]) are also publicly available. Raw data and uncropped blots associated with Figs. 1–6 and Supplementary Figs. 1–7 are provided as a single Source Data file. Patient sample information, metabolomics and lipidomics data, as well as oligonucleotide information are available in the Supplementary Files.

Field-specific reporting

Please select the one below that is the best fit for your research. If you are not sure, read the appropriate sections before making your selection.

☒ Life sciences ☐ Behavioural & social sciences ☐ Ecological, evolutionary & environmental sciences

For a reference copy of the document with all sections, see nature.com/documents/nr-reporting-summary-flat.pdf

Life sciences study design

All studies must disclose on these points even when the disclosure is negative.

Sample size	N = number of biological replicates is included in figure legends. For in vitro studies, at least three biological replicates were included. Sample size was determined to be adequate based on the magnitude of measurable differences between groups, which was established from previous published works (Tao et al., Adv. Sci. 2021, Moreno-Smith et al., Nat. Commun. 2021). For in vivo studies, sample size was calculated based on our previous work (Moreno-Smith et al., Nat. Commun. 2021). At least six mice per group are required to reach 80% statistical power at $p < 0.05$ (https://www.stat.ubc.ca/~rollin/stats/ssize/n2).
Data exclusions	Non-engrafted mice were excluded for data collection. For ORO and immunohistochemical staining, samples with poor staining quality or without tumor regions were excluded from ImageJ analysis.
Replication	Experiments were repeated at least three times (see n = number of biological replicates in figure legends). All attempts at replication were successful.
Randomization	For in vitro experiments, samples were randomly assigned to control or experimental groups. For in vivo experiments, mice were randomized and evenly allocated into treatment groups.
Blinding	Investigators were blinded in metabolomics, lipidomics, FA profiling, and pathological analyses. Investigators were not blinded to group allocation during data collection and/or analysis for the rest of experiments in this study. This is because the investigators directly conducted the experiments and collected data for the analyses.

Reporting for specific materials, systems and methods

We require information from authors about some types of materials, experimental systems and methods used in many studies. Here, indicate whether each material, system or method listed is relevant to your study. If you are not sure if a list item applies to your research, read the appropriate section before selecting a response.

Materials & experimental systems

n/a	Involved in the study	
☐	☒	Antibodies
☐	☒	Eukaryotic cell lines
☒	☐	Palaeontology and archaeology
☐	☒	Animals and other organisms
☐	☒	Human research participants
☒	☐	Clinical data
☒	☐	Dual use research of concern

Methods

n/a	Involved in the study	
☒	☐	ChIP-seq
☒	☐	Flow cytometry
☐	☒	MRI-based neuroimaging

Antibodies

Antibodies used

1) Rabbit polyclonal anti-MYCN, Cell Signaling Technology, Cat# 9405S; RRID: AB_10692664; 1:500 dilution
2) Mouse polyclonal anti-FATP1, Abcam, Cat# ab69458; RRID: AB_1270734; 1:500 dilution
3) Rabbit polyclonal anti-FATP2, Abcam, Cat# ab83763; RRID: AB_1859828; 1:500 dilution
4) Rabbit polyclonal anti-FATP2, Thermo Fisher Scientific, Cat# PA5-30420; RRID: AB_2547894; 1:500 dilution
5) Rabbit polyclonal anti-FATP2, Thermo Fisher Scientific, Cat# PA5-42429; RRID: AB_2610399; 1:500 dilution
6) Mouse monoclonal anti-total and cleaved PARP, BD Biosciences, Cat# 551024, clone# 7D3-6; RRID: AB_394008; 1:500 dilution
7) Mouse monoclonal anti-total caspase-3, Santa Cruz, Cat# sc-65497, clone# 4.1.18; RRID: AB_1120001; 1:500 dilution
8) Rabbit polyclonal anti-cleaved caspase-3, Cell Signaling, Cat# 9661S; RRID: AB_2341188; 1:300 dilution
9) Rabbit monoclonal anti-p21 (Waf1/Cip1), Cell Signaling, Cat# 2947S, clone# 12D1; RRID: AB_823586; 1:300 dilution
10) Mouse monoclonal anti-p53, Santa Cruz, Cat# sc-126, clone# DO-1; RRID: AB_628082; 1:500 dilution
11) Rabbit polyclonal anti-SCD1, Cell Signaling, Cat# 2438S; RRID: AB_823634; 1:1000 dilution

12) Rabbit polyclonal anti-ACC, Cell Signaling, Cat# 3662S; RRID: AB_2219400; 1:1000 dilution
13) Mouse monoclonal anti-CypB, Santa Cruz, Cat# sc-130626, clone# k2E2; RRID: AB_2169421; 1:500 dilution
14) Mouse monoclonal anti-ACTB, Sigma, Cat# A2228, clone# AC-74; RRID: AB_476697; 1:5000 dilution
15) IRDye® 680RD Goat anti-mouse IgG, LI-COR Biosciences, Cat# 925-68070; RRID: AB_2651128; 1:10000 dilution
16) IRDye® 800CW Goat anti-mouse IgG, LI-COR Biosciences, Cat# 926-32210; RRID: AB_621842; 1:10000 dilution
17) IRDye® 800CW Goat anti-Rabbit IgG, LI-COR Biosciences, Cat# 926-32211; RRID: AB_621843; 1:10000 dilution
18) Rabbit monoclonal anti-Ki67, Biocare Medical, Cat# CRM325A, clone# SP6; RRID: AB_2721189; 1:50 dilution
19) Mouse monoclonal anti-Ki67, Agilent, Cat# M7240, clone# MIB1; RRID: AB_2142367; 1:50 dilution
20) Rabbit polyclonal anti-cleaved caspase-3, Cell Signaling, Cat# 9661L; RRID: AB_2341188; 1:400 dilution
21) Mouse monoclonal anti-MYCN, Sigma, Cat# OP13-100UG, clone# NCM II 100; RRID: AB_213284; 1:100 dilution
22) Goat biotinylated anti-rabbit IgG, Vector Laboratories, Cat# BA1000; RRID: AB_2313606; 1:200 dilution
23) Goat biotinylated anti-mouse IgG, Vector Laboratories, Cat# BA9200; RRID: AB_2336171; 1:200 dilution
24) Mouse monoclonal anti-MYCN, Santa Cruz, Cat# sc-53993, clone# B8.4.B; RRID: AB_831602; 5 ug per IP
25) Rabbit polyclonal anti-C-Myc, Santa Cruz, Cat# sc-764, clone# N-262; RRID: AB_631276; 5 ug per IP

Validation

1) Anti-MYCN antibody (Cell Signaling, Cat# 9405S) was validated by western blot analysis of extracts from IMR32 neuroblastoma cells.
2) Anti-FATP1 antibody (Abcam, Cat# ab69458) was validated by western blot analysis of FATP1 transfected 293T lysate.
3) Anti-FATP2 antibody (Abcam, Cat# ab83763) was validated by western blot analysis of HeLa cell lysate.
4) Anti-FATP2 antibody (Thermo Fisher Scientific, Cat# PA5-30420) was validated by western blot analysis of rat tissue extracts and mouse liver lysate.
5) Anti-FATP2 antibody (Thermo Fisher Scientific, Cat# PA5-42429) was validated by western blot analysis of human Hela, Jurkat, PANC1 and MCF7 cell lysates.
6) Anti-total and cleaved PARP antibody (BD Biosciences, Cat# 551024) was validated by western blot analysis of Jurkat cell lysate.
7) Anti-total caspase-3 antibody (Santa Cruz, Cat# sc-65497) was validated by western blot analysis of CCRF-CEM, CCRF-HSB-2 and Jurkat cell lysates.
8) Anti-cleaved caspase-3 antibody (Cell Signaling, Cat# 9661S) was validated by western blot analysis of extracts from HeLa, NIH/3T3 and C6 cells untreated, staurosporine-treated (3hrs, 1 µM in vivo) or cytochrome c-treated (1hr, 0.25 mg/ml in vitro) as well as by immunohistochemical analysis of paraffin-embedded human tonsil.
9) Anti-p21 (Waf1/Cip1) antibody (Cell Signaling, Cat# 2947S) was validated by western blot analysis of control HeLa cells or p21 (Waf1/Cip1) knockout HeLa cells.
10) Anti-p53 antibody (Santa Cruz, Cat# sc-126) was validated by western blot analysis of A-431, SW480, A549 and HUV-EC-C cell lysates.
11) Anti-SCD1 antibody (Cell Signaling, Cat# 2438S) was validated by western blot analysis of 3T3-L1 cell lysates.
12) Anti-ACC antibody (Cell Signaling, Cat# 3662S) was validated by western blot analysis of extracts from 293, HeLa, A431, NIH/3T3, L929, C6, H-4-IIE and BAEC cells.
13) Anti-CypB antibody (Santa Cruz, Cat# sc-130626) was validated by western blot analysis of Hep G2, HeLa and HEK 293T cell lysates.
14) Anti-ACTB antibody (Sigma, Cat# A2228) was validated by western blot analysis of HeLa, Jurkat, COS7, NIH-3T3, PC-12, RAT2, CHO, MDBK, and MDCK cell lysates. Manufacturer also cites validations in mouse embryonic fibroblasts (in "Tao Y, Xi S, Briones V, Muegge K. Lsh mediated RNA polymerase II stalling at HoxC6 and HoxC8 involves DNA methylation. PLoS One. 2010 Feb 11;5(2):e9163. doi: 10.1371/journal.pone.0009163. PMID: 20161795; PMCID: PMC2820093") and mouse osteoblasts (in "Schulze J, Seitz S, Saito H, Schneebauer M, Marshall RP, Baranowsky A, Busse B, Schilling AF, Friedrich FW, Albers J, Spiro AS, Zustin J, Streichert T, Ellwanger K, Niehrs C, Amling M, Baron R, Schinke T. Negative regulation of bone formation by the transmembrane Wnt antagonist Kremen-2. PLoS One. 2010 Apr 27;5(4):e10309. doi: 10.1371/journal.pone.0010309. PMID: 20436912; PMCID: PMC2860505").
15) The IRDye® 680RD Goat anti-mouse IgG antibody (LI-COR Biosciences, Cat# 925-68070) is described on the manufacturer website as "tested by dot blot and and/or solid-phase adsorbed for minimal cross-reactivity with human, rabbit, goat, rat, and horse serum proteins, but may cross-react with immunoglobulins from other species. The conjugate has been specifically tested and qualified for Western blot and In-Cell Western™ assay applications.
16) The IRDye® 800CW Goat anti-mouse IgG antibody (LI-COR Biosciences, Cat# 926-32210) is described on the manufacturer website as "tested by dot blot and and/or solid-phase adsorbed for minimal cross-reactivity with human, rabbit, goat, rat, and horse serum proteins, but may cross-react with immunoglobulins from other species. The conjugate has been specifically tested and qualified for Western blot applications.
17) The IRDye® 800CW Goat anti-Rabbit IgG antibody (LI-COR Biosciences, Cat# 926-32211) is described on the manufacturer website as "tested by dot blot and and/or solid-phase adsorbed for minimal cross-reactivity with human, mouse, rat, sheep, and chicken serum proteins, but may cross-react with immunoglobulins from other species. The conjugate has been specifically tested and qualified for Western blot and In-Cell Western™ assay applications.
18) Anti-Ki67 antibody (Biocare Medical, Cat# CRM325A) was validated by immunohistochemical analysis of breast cancer tissue.
19) Anti-Ki67 antibody (Agilent, Cat# M7240) was validated by immunohistochemical analysis of high grade lymphoma tissue.
20) Anti-cleaved caspase 3 antibody (Cell Signaling, Cat# 9661L) was validated in immunohistochemical analysis of paraffin-embedded human tonsils, jurkat cells, and mouse embryo.
21) Anti-MYCN antibody (Sigma, Cat# OP13-100UG) was validated by immunohistochemical analysis of human neuroblastoma tissue (Wang et al., British Journal of Cancer, 2015).
22) Goat biotinylated anti-rabbit IgG antibody (Vector Laboratories, Cat# BA1000) was validated by immunohistochemical analysis of mouse kidney (van Swelm et al., Scientific Reports, 2022).
23) Goat biotinylated anti-mouse IgG antibody (Vector Laboratories, Cat# BA9200) was validated by immunohistochemical analysis of mouse brain (Puigdellívol et al., Cell Reports, 2021).
24) Anti-MYCN antibody (Santa Cruz, Cat# sc-53993) was validated by chromatin immunoprecipitation and sequencing of mouse prostate tumor tissue (Brady et al., Nat Commun, 2021).
25) Anti-C-Myc antibody (Santa Cruz, Cat# sc-764) was validated by chromatin immunoprecipitation and sequencing of mouse medulloblastoma tumor tissue (Vo et al., Cancer Cell, 2016).

General antibody quality and validation statements are provided by each of the following antibody manufacturers:
Cell Signaling: "To ensure product performance, we validate all of our antibodies, in-house, in multiple research applications."
Abcam: "We promise guaranteed product quality and expert customer service" and "Our Abpromise guarantee covers product applications & species that have been tested in our own labs, by our suppliers, or by selected trusted collaborators"

Thermo Fisher Scientific: "Each antibody is manufactured with high quality to enable customers to obtain reproducible results. Each antibody is designed to meet the applicable specification as stated on the product data sheets, including but not limited to species reactivity, application suitability, and detection of target of interest as identified by the stated UniProt ID."
BD Biosciences: "At BD Biosciences, we pride ourselves on our excellent product and technical support. All our products are backed by our >45 years of flow cytometry expertise and support resources."
Santa Cruz: "Santa Cruz Biotechnology is committed to providing the highest level of quality and service. Any product that does not meet the performance standards indicated in our product literature will be replaced at no charge."
Sigma: "We promise to offer industry leading quality antibodies supported by exemplary scientific support and we want to empower you to explore the biology of the world around us with no risk" and "Our development and manufacturing processes are subject to rigorous quality control and quality assurance measures, and each of our antibody products is supplied with a comprehensive Certificate of Analysis and Product Information Sheet."
Biocare Medical: "Biocare Medical's vast portfolio of over 300 antibodies meets your clinical and research laboratory needs. Choose from a wide variety of offerings within each antibody from concentrate, ready to use predilute or pre-optimized antibodies built to operate on various automated instruments such as the ONCORE Pro, intelliPATH FLX, Leica Bond, or Roche Benchmark Ultra. Biocare is your solution for top-performing, high-quality antibodies."
Agilent: "Dako's manufacturing facilities in Denmark and the USA have established quality management systems designed to live up to the most stringent standards in the in vitro diagnostics industry."
Vector Laboratories: "Vector Laboratories provides only the highest quality products that are rigorously tested in the intended applications. Our knowledgeable technical staff is ready to assist in the successful use of our reagents in a myriad of applications."

Eukaryotic cell lines

Policy information about cell lines

Cell line source(s)

The human NB cell lines IMR32 (male, RRID: CVCL_0346, CCL-127), SK-N-AS (female, RRID: CVCL_1700, CRL-2137), SH-SY5Y (female, RRID: CVCL_0019, CRL-2266) were purchased from ATCC.
SHEP (female, RRID: CVCL_0524) and MYCN3 cells were provided by Dr. Jason M. Shohet lab, University of Massachusetts. No commercial source.
Kelly (female, RRID: CVCL_2092, Sigma 92110411), SK-N-BE(2c) (male, RRID: CVCL_0529, ATCC CRL-2268), and LAN5 shMYCN cells were provided by Dr. Ronald Bernardi lab, Baylor College of Medicine, BCM.
LAN5 cells (male, RRID: CVCL_0389) were provided by Dr. Leonid S. Metelitsa lab, BCM. No commercial source
SK-N-AS MYCN-ER™ cells were provided by Dr. Brian J. Altman lab, University of Rochester. No commercial source.
HS-5 bone marrow stroma cells (male, RRID: CVCL_3720, ATCC CRL-11882) were provided by Dr. Michele Redell lab, Texas Children's Hospital.
C2C12 mouse myoblast cells (female, RRID: CVCL_0188, ATCC CRL-1772) were provided by Dr. Joel R Neilson lab, BCM.
ARPE-19 retinal pigmented epithelial cells (male, RRID: CVCL_0145, ATCC CRL-2302) were provided by Dr. Huda Zoghbi lab, BCM.
Tet21/N cells (female, RRID: CVCL_9812) were provided by Dr. Giovanni Perini lab, University of Bologna. No commercial source.
IMR32 cells with inducible FATP1 or FATP2 expression were generated in the lab. No commercial source.
LAN5/IMR32/SK-N-AS shCTRL and shSLC27A2 cells were generated in the lab. No commercial source.

Authentication

All cell lines were validated by STR analysis.

Mycoplasma contamination

All cell lines were negative for mycoplasma.

Commonly misidentified lines
(See ICLAC register)

No common misidentified lines were used in the study.

Animals and other organisms

Policy information about studies involving animals; ARRIVE guidelines recommended for reporting animal research

Laboratory animals

Female and male six-week-old TH-MYCN+/+ mice (129x1/svj); female six-week-old TH-MYCN-/- mice (129x1/svj); female six-week-old NCr nude mice (CrTac:NCr-Foxn1nu), female and male six to eight-week-old NOG mice (NOD.Cg-Prkdcscid Il2rgtm1Sug/JicTac). Mice were housed at the TCH Animal Facility with a temperature (21±1oC) and humidity (60%)-controlled and specific pathogen-free environment under a 14 h:10 h light/dark cycle. Mice were fed standard chow diet (LabDiet, 3002906-704) ad libitum.

Wild animals

No wild animals were used.

Field-collected samples

No field-collected samples were used.

Ethics oversight

In vivo studies were approved by the BCM Institutional Animal Care and Use Committee (AN7089 and AN6190).

Note that full information on the approval of the study protocol must also be provided in the manuscript.

Human research participants

Policy information about studies involving human research participants

Population characteristics

Frozen primary tumors (MYCN-amplified, MNA, n = 18; non MYCN-amplified, non-MNA, n = 18) were provided by the Research Tissue Support Service (RTSS) at Texas Childrens' Hospital (TCH) . Patient clinical information is summarized in Supplementary Data 1.

Recruitment	No recruitment in this study.
Ethics oversight	The study was approved by the Institutional Review Board for Human Subject Research for Baylor College of Medicine and Affiliated Hospitals (BCM IRB, H-42596, H-6650). Informed consent was obtained from all participants. Compensation was not provided.

Note that full information on the approval of the study protocol must also be provided in the manuscript.

Magnetic resonance imaging

Experimental design

Design type	Anatomical/Structural Imaging - non-invasive assessment of tumor growth
Design specifications	MRI was performed on day 1 and day 14 of treatment to monitor tumor growth.
Behavioral performance measures	No behavioral performance measures were used in these studies.

Acquisition

Imaging type(s)	Anatomical/Structural
Field strength	1T
Sequence & imaging parameters	T2-weighted fast spin echo - TR: 3030 ms, TE: 80 ms, Slice Thickness: 1.2 mm, FOV: 80 mm, Matrix: 256x250, NEX = 2, Dwell time = 25 us, Scan Time = 2.56 min
Area of acquisition	Coronal slices were taken through the mouse abdomen

Diffusion MRI ☐ Used ☒ Not used

Preprocessing

Preprocessing software	No preprocessing was used in this study.
Normalization	As only structure/anatomy was assessed, no MRI signal intensities/data were normalized in this study.
Normalization template	As only structure/anatomy was assessed, no MRI signal intensities/data were normalized in this study.
Noise and artifact removal	No denoising methods were used in this study.
Volume censoring	No volume censoring was used in this study.

Statistical modeling & inference

Model type and settings	No statistical modeling was used in these MRI volumetric studies
Effect(s) tested	No statistical modeling was used in these MRI volumetric studies

Specify type of analysis: ☐ Whole brain ☒ ROI-based ☐ Both

Anatomical location(s)	Tumors were manually segmented in the abdominal field of view from surrounding organs (e.g. kidneys, liver, and spleen). The segmented volumes for each subject were used for analysis.
Statistic type for inference (See Eklund et al. 2016)	Voxel-wise
Correction	No corrections to the images or ROIs were used in this study.

Models & analysis

n/a	Involved in the study
☒	☐ Functional and/or effective connectivity
☒	☐ Graph analysis
☒	☐ Multivariate modeling or predictive analysis
